# Hydrogen Sulfide Inhibits L-Type Calcium Currents Depending upon the Protein Sulfhydryl State in Rat Cardiomyocytes

**DOI:** 10.1371/journal.pone.0037073

**Published:** 2012-05-10

**Authors:** Rongyuan Zhang, Yan Sun, Haojan Tsai, Chaoshu Tang, Hongfang Jin, Junbao Du

**Affiliations:** 1 Department of Pediatrics, Peking University First Hospital, Beijing, People's Republic of China; 2 Department of Central Laboratory, Peking University First Hospital, Beijing, People's Republic of China; 3 Institute of Cardiovascular Research, Peking University First Hospital, Beijing, People's Republic of China; 4 Key Laboratory of Molecular Cardiovascular Diseases, Ministry of Education, Beijing, People's Republic of China; University of Southern California, United States of America

## Abstract

Hydrogen sulfide (H_2_S) is a novel gasotransmitter that inhibits L-type calcium currents (I _Ca, L_). However, the underlying molecular mechanisms are unclear. In particular, the targeting site in the L-type calcium channel where H_2_S functions remains unknown. The study was designed to investigate if the sulfhydryl group could be the possible targeting site in the L-type calcium channel in rat cardiomyocytes. Cardiac function was measured in isolated perfused rat hearts. The L-type calcium currents were recorded by using a whole cell voltage clamp technique on the isolated cardiomyocytes. The L-type calcium channel containing free sulfhydryl groups in H9C2 cells were measured by using Western blot. The results showed that sodium hydrosulfide (NaHS, an H_2_S donor) produced a negative inotropic effect on cardiac function, which could be partly inhibited by the oxidant sulfhydryl modifier diamide (DM). H_2_S donor inhibited the peak amplitude of I_ Ca, L_ in a concentration-dependent manner. However, dithiothreitol (DTT), a reducing sulfhydryl modifier markedly reversed the H_2_S donor-induced inhibition of I _Ca, L_ in cardiomyocytes. In contrast, in the presence of DM, H_2_S donor could not alter cardiac function and L type calcium currents. After the isolated rat heart or the cardiomyocytes were treated with DTT, NaHS could markedly alter cardiac function and L-type calcium currents in cardiomyocytes. Furthermore, NaHS could decrease the functional free sulfhydryl group in the L-type Ca^2+^ channel, which could be reversed by thiol reductant, either DTT or reduced glutathione. Therefore, our results suggest that H_2_S might inhibit L-type calcium currents depending on the sulfhydryl group in rat cardiomyocytes.

## Introduction

In addition to the gasotransmitters nitric oxide (NO) and carbon monoxide (CO), hydrogen sulfide (H_2_S) is the third biologic signal gaseous molecule and is recognized as an important physiologic regulator in the circulatory, nervous, endocrine and immune systems [1]. In the investigation of broad physiological functions, the cardio-protective effect of H_2_S was first found and drew much attention in the field of life sciences. H_2_S can be endogenously generated from cysteine by the cystathionine-Υ-lyase (CSE) enzyme in the cardiovascular system [2]. *In vitro* and *in vivo* experiments showed that H_2_S induced negative cardiac inotropic effects and played a cardio-protective role in various models of diseases. It was also found that exogenous H_2_S post-conditioning successfully protected isolated rat hearts against ischemia-reperfusion injury [3] and played a protective role in chronic heart failure [4]. However, the mechanism responsible for the negative cardiac inotropic effects of H_2_S has not been fully understood.

L-type calcium channels are decisive in the excitation/contraction coupling in cardiomyocytes, and they provide the main pathway through which Ca^2+^ enters into myocardial cells; therefore, the Ca^2+^ entering through these channels may trigger the Ca^2+^-induced Ca^2+^ release. The amount of Ca^2+^ released from intracellular calcium stores and the Ca^2+^ entering the sarcoplasmic reticulum (SR) from outside the cells maintain intracellular calcium homeostasis, which plays a fundamental role in myocardial physiology and pathology [5]. In 2008, Sun, et al. demonstrated that H_2_S could inhibit L-type calcium channels in cardiomyocytes [6]. However, the potential targeting site on L-type calcium channels has not been clarified.

H_2_S is more potently toxic than cyanide since it blocks cytochrome C oxidase that results in mitochondrial respiration inhibition [7], [8]. The transformation of disulfide bridges into sulfhydryl groups of the cysteine-containing proteins at the center of cytochrome C oxidase was regarded as the mechanism for intoxication of H_2_S [9]. Toxicological experiments showed that pre-treatment with oxidized glutathione (GSSG) or methemoglobinemia could protect experimental mammals against a subsequent lethal challenge from inorganic sulfide poisoning; alternatively, a method of de-intoxication of H_2_S involves trapping free sulfide which may prevent it from reaching a vital enzymatic site [9]. Thus, the disulfide bridges or the sulfhydryl groups of the cysteine-containing proteins may be the effective targets of H_2_S. Meanwhile, the subunits of the L-type calcium channel [10] and ATP sensitive potassium channel [11] were found to contain functionally important free sulfhydryl groups that modulate gating. Therefore, we hypothesized that a novel mechanism of activation of the channels might resulted from the formation of a disulfide bridge between cysteine residues of the pore and that H_2_S might have an accommodating gate on the channels mentioned above with “Cys-SH” as the critical target.

The protein structure and function of thiol-containing compounds, containing cysteine residues which can form a disulfide bond when the sulfhydryl group of cysteine is oxidized, could be altered. Sulfhydryl reagents have been widely used as a pharmacological tool to explore the molecular functions of channel proteins. The fact that L-type calcium channels are subjected to direct modification by sulfhydryl reagents has been demonstrated [12].

Therefore, the present study was undertaken to investigate whether the inhibitory effects of L-type calcium channel induced by H_2_S was dependent on the disulfide bridge or sulfhydryl group.

## Methods

### Ethics Statement

All animal experimental procedures conformed to the “Guide for the Care and Use of Laboratory Animals” published by the National Institutes of Health (NIH) in the United States and “The use of non-human primates in research”, and the Animal Research Ethics Committee of Peking University First Hospital specifically approved this study with the permit number of J200913.

### Animals

Male Sprague-Dawley (SD) rats with a body weight of 200–250 g were obtained from Vital River (Beijing, China). The rats were housed in cages and fed a standard laboratory diet and fresh water. The cages were kept in a room with controlled temperature (24±1°C), relative humidity (65–70%) and 12 hour light/dark cycle.

### Chemicals

NaHS, collagenase I, protease E aminoethylsulfonic acid, L-aminoglutaminic acid, CsOH, CsCl, nifedipine, (±) Bay K8644, diamide (DM), dithiothreitol (DTT), reduced L-glutathione (GSH), L-cysteine (L-CY), Na_2_ATP, and Na_2_GTP were purchased from Sigma (St Louis, MO, USA). Bovine serum albumin (BSA), HEPES and EGTA were purchased from Amresco (Solon, USA). TTX was purchased from Aquatic Products Research Institute (Hebei, China). NaHS was dissolved in bath solutions. Fresh stock solutions were then diluted with bath solution to yield H_2_S solutions of various concentrations.

### Experimental protocol of measurement of cardiac function *in vivo*

All rats were anesthetized with 12% urethane (1 ml/100 g, i.p.). The isolated hearts were removed quickly and fixed using the Langendorff perfusion apparatus with the left auricular appendage removed. They were retroperfused in the aorta with the 37°C Krebs-Henseleit (K-H) solution containing the following at mmol/L concentrations: NaCl, 118.0; KCl, 4.7; KH_2_PO_4_, 0.93; MgSO_4_·7H_2_O, 1.2; CaCl_2_, 1.5; NaHCO_3_, 25; C_6_H_12_O_6_, 11.0; pH 7.4, mixed by 95% O_2_ and 5% CO_2_. A balloon catheter was inserted into the left ventricle for the measurement of left ventricular systolic pressure (LVSP) and the left ventricular (LV) pressure (±dp/dt_max_). The balloon was connected to a pressure transducer with the computer. The fluid was adjusted to obtain a left ventricular end-diastolic pressure (LVEDP) under 10 mmHg. For all rats, cardiac function was assessed by using the Powerlab (4S, Australia) after a 20 min equilibration period. Subsequent procedures were as follows. Thirty-three rats were randomly divided into five groups: 1) isolated rat hearts (n = 6) were equilibrated 20 min in the K-H solution, then perfused with the K-H solution with 100 µmol/L NaHS for 10 min, and the cardiac function was again determined by Powerlab; 2) after 20 min stabilization, the isolated hearts (n = 6) were perfused with the K-H solution with 100 µmol/L DM for 5 min, and the cardiac function of this stage was also recorded. Subsequently, the K-H solution with 100 µmol/L NaHS was used to perfuse the hearts and the data were assessed; 3) isolated rat hearts (n = 6) were firstly equilibrated 20 min in the K-H solution, and then perfused with the K-H solution with 100 µmol/L DTT for 5 min. Finally the K-H solution with 100 µmol/L NaHS was infused into the hearts, and the cardiac functions were observed by Powerlab; 4) isolated rat hearts (n = 9) were perfused with the K-H solution with nifedipine at a dosage of 10 µmol/L for 5 min, and the cardiac function at this stage was recorded. Subsequently, hearts were perfused with the K-H solution with 100 µmol/L NaHS, and the data were also recorded; 5) isolated rat hearts (n = 6) were perfused with the K-H solution with nifedipine at a dosage of 10 µmol/L for 5 min, and the cardiac function was recorded at this stage. The hearts were subsequently perfused with the K-H solution alone and the same indexes were recorded by Powerlab. Alteration of left ventricular pressure [ΔLVP = left ventricular systolic pressure (LVSP)-left ventricular end diastolic pressure (LVEDP)] was calculated to reflect the maximum contractility of left ventricle myocardium; +dp/dt_max_ indicates the maximum contractile velocity of myocardium, while −dp/dt_max_ represents the myocardial maximum diastolic ability.

### Cardiomyocyte isolation

Single cells were obtained by following a method described by Zhang *et al.* with modifications [13]. Briefly, each rat was anesthetized with 12% ethylcarbamate (1 ml/100 g i.p.). The heart was rapidly excised and attached to an improved Langendorf perfusion apparatus. The heart was then retrogradely perfused for 5 min at 37°C with Ca^2+^-free Tyrode's solution containing (in mmol/L) NaCl 137, KCl 5.4, NaH_2_PO_4_ 0.33, MgCl_2_ 1.0, glucose 10, and HEPES 10, and the pH was adjusted to 7.3–7.4 with NaOH after the solution was equilibrated with 95% O_2_ and 5% CO_2_. Enzymatic digestion with a steady perfusion pressure of 80 mmol/L Hg was achieved by re-circulating the perfusion apparatus with the low calcium oxygenated Tyrode's solution containing 0.8 mg/ml collagenase Type I, protease E 0.1 mg/ml, 0.5 mg/ml BSA, and 20 µmol/L Ca^2+^ for 13–15 min. The ventricles were separated from the heart, cut into small pieces, and stirred to obtain a cell suspension at 37°C in oxygenated KB solution containing (in mmol/L) KOH 80, KCl, 40, KH_2_PO_4_ 20, glutamic acid 50, MgSO_4_ 3, taurine 20, EGTA 0.5, HEPES 10, and glucose 10, and the pH was adjusted to 7.3–7.4 with KOH. After 3 min of stirring for 3 separate times, the cell suspensions were centrifuged and washed with 1 mmol/L CaCl_2_. Finally, the isolated cells were suspended in KB solution containing 0.5 mg/ml BSA and stored at room temperature for 30 min to 1 h before experiments. Rod-shaped cells with clear cross-striations without automatic contraction were used in the present study.

### Voltage-clamp recording

Currents of L-type calcium channels were recorded under voltage clamping in the whole-cell configuration of the patch-clamp technique. Cardiomyocytes were placed in a dish at the stage of an inverted microscope (IX70, Olympus Inc., Tokyo, Japan) and were continuously perfused at a constant rate (1.5 ml/min) with a oxygenated solution containing (in mmol/L) NaCl 137, CaCl_2_ 1.8, MgCl_2_ 1, CsCl 5.4, TTX 0.02, 4-AP 4, HEPES 10, and glucose 10 (pH adjusted to 7.3–7.4 with NaOH). Single cells were voltage-clamped using a patch-clamp amplifier (Axopatch 200B, Axon Instruments, Burlingham, CA, USA). Physiological signals was recorded by pClamp 6.0 (Axon Instruments). Pipettes for whole-cell patch-clamp recordings were made from borosilicate glass capillaries and had resistances of 1 to 3 MΩ. The pipette solution contained (in mmol/L) CsCl 130, MgCl_2_ 1, Na_2_ATP 5, Na_2_GTP 0.5, EGTA 11, and HEPES 10 (pH adjusted to 7.3 with CsOH). The I _Ca, L_ current was measured under the conditions described above. K^+^ currents were suppressed by internal Cs^+^ and 4-AP in the perfusion solution, as well as by external K^+^-free solution. The Na^+^ current was suppressed by TTX. The Na^+^-K^+^ pump current was inactivated in K^+^-free bath solutions and Na^+^-free pipette solutions. Membrane currents associated with Na^+^-Ca^2+^ exchange was eliminated by the Na^+^-free and low-Ca^2+^ (11 mmol/L EGTA) pipette solutions. Application of nifedipine (10 µmol/L) to the bath solution could completely inhibit the peak I _Ca, L_ within 1 min, confirming that the measured current was due to I _Ca, L_.

I–V relationship of I _Ca, L_ was obtained by plotting the peak current amplitude in response to voltage pulses to potentials between −40 and +70 mV from a holding potential of −40 mV (steps of 10 mV increments). The steady state activation of I _Ca, L_ was determined by applying 200 ms of depolarizing pulses between −70 mV and +30 mV from a holding potential of −70 mV. The steady-state inactivation of I _Ca, L_ was determined by applying a two-pulse protocol containing 1 s pre-pulses between −70 and +30 mV and a subsequent 200 ms of test pulse to 0 mV from a holding potential of −70 mV. The recovery of I _Ca, L_ from inactivation was tested with a double-pulse protocol consisting of a 200 ms of conditioning pulse to 0 mV followed by a 200 ms of test pulse to 0 mV from a holding potential of −70 mV with increasing interval steps of 20 ms between 20–500 ms. To standardize membrane currents to Cm, the capacity current transiently measured in response to a 5 mV hyperpolarizing pulse was integrated and divided by the given voltage to yield total Cm for each cell. Various concentrations of NaHS were applied by a fast puffing system. All experiments were performed at a room temperature of 21–23°C.

### Cell culture and identification of protein containing free sulfhydryl groups

H9C2 cells grown in 100-mm plates were incubated with Dulbecco's modified Eagle's medium (DMEM, Invitrogen, Carlsbad, CA, USA) administrated with 10% fetal bovine serum (FBS, Invitrogen, Carlsbad, CA, USA), 2 mmol/L L-glutamine, 100 U/ml penicillin and 100 µg/ml streptomycin under 37°C, 5% CO_2_ and saturated humidity for cell growth. When H9C2 cells reached at 80–90% confluence, the complete medium was changed into serum-free medium for 24 h and then incubated in DMEM containing 10% FBS. In the NaHS group, H9C2 cells were respectively treated with NaHS at 100 µmol/L for 30 min, DM at 100 µmol/L for 5 min, DTT at 5 mmol/L for 5 min, GSH at 5 mmol/L for 5 min, L-CY at 5 mmol/L for 5 min, and NaHS at 100 µmol/L for 25 min followed by DTT at 5 mmol/L for 5 min or followed by GSH at 5 mmol/L for 5 min. While, in the control group H9C2 cells were just incubated with 10% FBS DMEM for the same period. Then, H9C2 cells were solubilized in 1 ml of lysis buffer, and cell lysates were incubated with 50 µl of EZ-LinkTM PEO-iodoacetyl Biotin (10 mg/ml; Pierce) for 12 h at 4°C and then incubated with 30 µl of UltraLinkTM Immobilized NeutrAvidinTM (Pierce) for 4 h on a roller system at 4°C. The beads were washed twice with 1 ml of lysis buffer and three times with 1 ml of PBS. For Western blot analysis, proteins containing sulfhydryl groups of H9C2 cells were subjected to SDS-PAGE, and the proteins were transferred to nitrocellulose membranes. Membranes were probed with anti-L-type calcium channel antibody (Sigma, Saint Louis, Missouri, USA) and developed with Western blotting luminol reagents (Santa Cruz Biotechnology, Santa Cruz, CA, USA).

### Statistical analysis

The data were analyzed with the pCLAMP 10.0 (Axon Instruments), SPSS 13.0 and Microcal Origin 6.0 software. All data in the figures were expressed as mean ± SD. Differences among groups were analyzed with one-way ANOVA followed by LSD or Dunnett's post-hoc test where applicable. Significance was established at the *P*<0.05 level.

## Results

### The effect of NaHS on cardiac function

**Figure 1:**
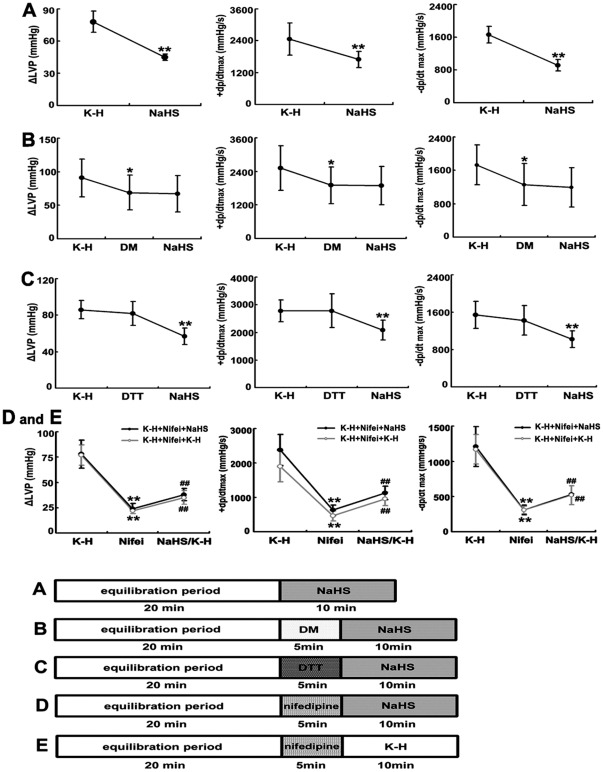
NaHS and sulfhydryl modifiers impacted NaHS-induced cardiac function. A: NaHS (100 µmol/L) depressed LV ± dp/dt_max_ and ΔLVP significantly as compared with the control. ^**^ *P*<0.01 vs. control. B: NaHS (100 µmol/L) could not change LV ± dp/dt_max_ and ΔLVP in the presence of DM perfusion. ^*^ *P*<0.05 vs. control, ^#^ *P*<0.05 vs. DM. C: NaHS (100 µmol/L) could depress LV ± dp/dt_max_ and ΔLVP in the presence of DTT. ^**^P<0.01 vs. DTT group. D and E: There were no significant differences in the change in the ventricular ±dp/dt_max_ and ΔLVP between the perfusate with and without NaHS following nifedipine perfusion (*P*>0.05). The gray line stands for the experiment protocol “K-H +Nifei+K-H”, and the black line stands for the experimental protocol “K-H +Nifei+NaHS”. ^**^ *P*<0.01 vs control group. ^##^ *P*<0.01 vs. nifedipine group.

With 100 µmol/L NaHS continuous perfusion at a physiological dosage for 10 min, LV ± dp/dt_max_ and ΔLVP decreased significantly compared with the control (*P*<0.01, Fig. 1A).

### Sulfhydryl modifiers impacted NaHS-induced inhibition of cardiac function in isolated perfused rat hearts

To examine if the NaHS-induced inhibitory effect on cardiac function in isolated perfused rat hearts depended upon the protein sulfhydryl group, we used DM, an oxidizing sulfhydryl modifier to transform protein sulfhydryl groups into disulfide bridges. The LV ±dp/dt_max_ and ΔLVP decreased after perfusion with DM at dosage of 100 µmol/L for 5 min as compared with controls (*P*<0.05, Fig. 1B). However, in the presence of DM perfusion fluid, the LV ±dp/dt_max_ and ΔLVP were not altered when continuously perfused with 100 µmol/L NaHS for 10 min (*P*>0.05, Fig. 1B).

Next, we used DTT, a reducing sulfhydryl modifier, in the perfusion fluid to see if it could mediate the inhibition of cardiac function induced by NaHS. In addition to the fact that LV ±dp/dt_max_ and ΔLVP did not change during perfusion with 100 µmol/L DTT for 5 min as compared with controls (*P*>0.05, Fig. 1C), we found that continuous perfusion of K-H solution with 100 µmol/L NaHS for 10 min in the presence of DTT obviously decreased the LV ±dp/dt_max_ and ΔLVP, compared to DTT perfusion without NaHS treatment (*P*<0.01, Fig. 1C).

### The effect of nifedipine on cardiac function in isolated perfused rat hearts treated by NaHS

Compared with controls, the LV ±dp/dt_max_ and ΔLVP decreased when perfused with the K-H solution consisting of nifedipine at a dosage of 10 µmol/L for 5 min (*P*<0.05, Fig. 1D and E). However, after continuous perfusion with the K-H solution for 10 min, the ventricular ±dp/dt_max_ and ΔLVP increased significantly as compared to those with K-H solution consisting of nifedipine (*P*<0.01, Fig. 1E). Furthermore, the data showed that continuous perfusion with NaHS at a dosage of 100 µmol/L following nifedipine perfusion could increase the ventricular ±dp/dt_max_ and ΔLVP (*P*<0.01). However, there were no significant differences in the change in the ventricular ±dp/dt_max_ and ΔLVP between the perfusate with and without NaHS following nifedipine perfusion (*P*>0.05, Fig. 1D and 1E). Those results suggested that pretreatment with nifedipine to inhibit L-Ca^2+^ channel could block the negative inotropic effect of NaHS.

### Characteristics of the L-type calcium channel current in rat ventricular cardiomyocytes

**Figure 2:**
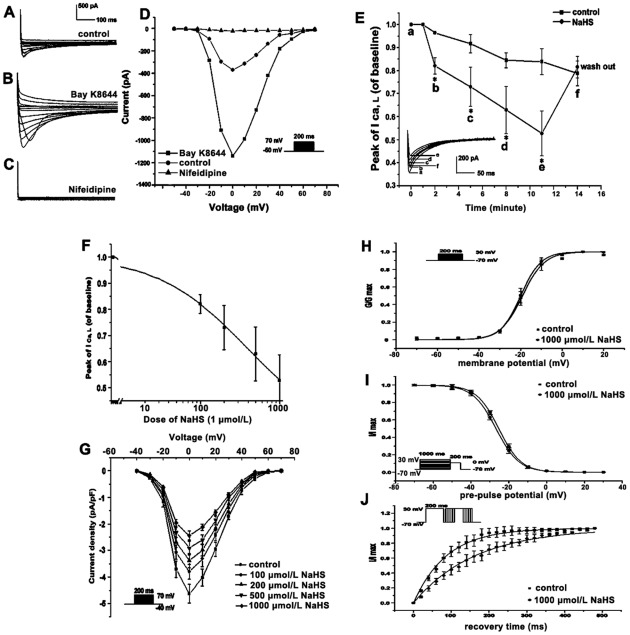
Representative L-type calcium current (I _Ca, L_) in rat ventricular cardiomyocytes (A, B, C and D); NaHS inhibits the peak of I _Ca, L_, and a gradual augmented concentration response relationship of NaHS-induced inhibition on I _Ca, L_ (E); NaHS inhibited I _Ca, L_ (F and H); and effect of NaHS on the kinetics of I _Ca, L_ activation and inactivation (I, G and K). A: Typical traces of whole-cell superimposed I _Ca, L_. B: I _Ca, L_ was enlarged by 1 µmol/L Bay K 8644. C: I _Ca, L_ was completely inhibited by 10 µmol/L nifedipine. D: Nifedipine could almost completely inhibit (95%) the inward current, and Bay K 8644 could increase the inward current markedly (300%). E: Application of increasing concentrations of NaHS (100, 200, 500 and 1000 µmol/L) significantly reduced the amplitude of the peak of I _Ca, L_, respectively, as shown in the figure labeled as b, c, d, and e, respectively (“a” stands for the beginning). The inhibition of I _Ca, L_ preceded rapidly in the first 1 min, and during the washout period (5 min) I _Ca, L_ could be partially recovered (n = 6 for each group). *P<0.05 vs. control. F: The inhibitory effects of NaHS on the peak of I _Ca, L_. Statistically significant decreases in currents were apparent in four separate concentrations of NaHS (100, 200, 500 and 1000 µmol/L)-treated cells. G: The mean current density-voltage for I _Ca, L_ in rat left ventricular cardiomyocytes decreased significantly by four separate concentrations of NaHS (100, 200, 500 and 1000 µmol/L). H: 1000 µmol/L NaHS did not change the steady-state activation curve of the L-type calcium channel. I: 1000 µmol/L NaHS did not change the steady-state inactivation curves of the L-type calcium channel. J: NaHS induced a shift in the kinetics of recovery of I _Ca, L_ from inactivation; and the I/I _max_ values of the NaHS-perfused group significantly decreased in comparison with those of the control.

The L-type calcium currents were activated by a series of depolarizing pulses from −50 mV to +70 mV at 10 mV increments. This inward current could be almost completely inhibited (95%) by 10 µmol/L nifedipine, a specific L-type calcium channel blocker, and could be increased markedly (300%) by 1 µmol/L Bay K 8644. Fig. 2A, B, C and D show the representative traces and the corresponding I–V curves. The peak of the I–V curve of the I _Ca, L_ was at membrane potentials of 0 mV under control conditions and bath application of 1 µmol/L Bay K 8644.

### Inhibitory effect of NaHS on I _Ca, L_ in rat ventricular cardiomyocytes

I _Ca, L_ was elicited by pulses from a holding potential of −40 mV to 0 mV for 200 ms every 1 min using the whole-cell patch clamp technique. Four increasing concentrations of NaHS (100, 200, 500 and 1000 µmol/L) were successively applied to the cell for 3 min duration of perfusion per concentration, and the effects of NaHS on the I _Ca, L_ were detected. Representative current traces in ventricular cardiomyocytes are shown in Fig. 2E. Application of increasing concentrations of NaHS (100, 200, 500 and 1000 µmol/L) significantly reduced the amplitude of the peak of I _Ca, L_ to 85.11±4.33%, 79.54±11.65%, 74.44±16.29% and 62.85±18.53% of the value in the control at the same time point, respectively. The inhibition of I _Ca, L_ preceded rapidly in the first 1 min, and during the washout period (5 min) I _Ca, L_ could be partially recovered. Thus, the effects of NaHS on I _Ca, L_ were reversible at least in part.

### Concentration-dependent inhibitory effect of NaHS on I _Ca, L_

As shown in Fig. 2F and H, the bath application of NaHS in various concentrations also inhibited the peak amplitude of the calcium current. The NaHS decreased the concentration-response curves of I _Ca, L_ evoked by a single pulse from −40 mV to 0 mV for 200 ms in the rat ventricular cardiomyocytes. In comparison with the control, the peak amplitude of calcium current was decreased successively from 82.09±3.55%, 72.97±8.51%, 62.91±10.25% to 52.75±9.78% of the control values by NaHS at concentrations from 100, 200, 500 through 1000 µmol/L, respectively. Dose-response curves were fitted by the logistic function: Y = (A_1_-A_2_)/[1+(x/x_0_)^p^]+A_2_, and Kd of NaHS on I _Ca, L_ was 376.66±21.78 µmol/L. Fig. 2F and H show the I–V curves constructed in the absence or presence of NaHS by applying a 200 ms voltage pulse ranging from −40 mV to +70 mV, in 10 mV increments. In order to avoid the influence of different cell sizes, the I _Ca-L_ was divided by the membrane capacitance, an index of cell surface area. From Fig. 2F and H, I_Ca, L_ density was decreased significantly in ventricular cardiomyocytes obtained from NaHS perfused groups (−2.44±0.17 pA/pF, −2.91±0.26 pA/pF, −3.37±0.22 pA/pF and −3.80±0.29 pA/pF for 1000, 500, 200 and 100 µM NaHS perfused groups, respectively) compared to those from the control (−4.63±0.34 pA/pF, n = 6, *P*<0.05). Application of NaHS showed a concentration-dependent suppression on the peak of the I–V curves without altering the reversal potential and the voltage dependence of peak I _Ca, L_.

### Effect of NaHS on the current kinetics of L-type calcium channel activation and inactivation

After perfusion of the cardiomyocytes with 1000 µmol/L NaHS, the steady-state activation curve of the L-type calcium channel (Fig. 2H) showed that the half-maximal activation voltage (V_1/2_) did not change (from −20.1±0.65 to −19.45±0.76 mV, *P*>0.05, n = 8). The K values were 4.85±0.47 and 5.27±0.69 in the control and NaHS treated groups (*P*>0.05), respectively, without shifting in the activation curve. For the steady-state activation curve, currents were elicited by applying a series of 200 ms of depolarizing pulses (range from −70 mV to +30 mV in 10 mV increments) from a holding potential of −70 mV, and the activation curves were fitted by the Boltzmann equation: G _Ca_/G _Ca Max_ = 1-{1+exp[-(Vm-V_1/2_)/k]}^−1^.

Meanwhile, the effects of NaHS on the steady-state inactivation characteristics of the L-type calcium channel (Fig. 2I) in ventricular cardiomyocytes were observed with a 200 ms test pulse of 0 mV after various pre-pulses which lasted for 1 s each (range: from −70 mV to +30 mV; in 10 mV increments) to a holding potential of −70 mV. The inactivation curves were calculated using the Boltzmann equation: I _Ca_/I _Ca max_ = {1+exp [(Vm-V_1/2_)/k]}^−1^. However, there was no significant difference in the inactivation characteristics of the L-type calcium channel between those of the NaHS perfused and of the control groups. V_1/2_ values were −25.38±0.68 and −25.84±0.59 mV in the control and the NaHS-treated groups (*P*>0.05, n = 8), respectively. The K values were 5.88±0.25 and 6.03±0.37 in the control and NaHS perfused groups, respectively. There was no significant shift in the steady state inactivation curve of I _Ca, L_.

The kinetics of recovery of I _Ca, L_ from the inactivation curves were tested with a double-pulse protocol: a 200 ms of conditioning pulse to 0 mV followed by a 200 ms of test pulse to 0 mV from the holding potential of −70 mV with increasing intervals to 500 ms in increments of 20 ms. The recovery curve could be fitted by the exponential equation: I _Ca_/I _Ca max_ = 1−exp (−t/τ). There was a significant extension of I _Ca, L_ recovery from inactivation, since the time constant (τ value) changed from 70.56±4.43 to 162.86±27.75 ms in the control and the NaHS (1000 µmol/L)- treated groups, respectively (*P*<0.01, n = 8) (Fig. 2J). The time course of the recovery from the inactivation of I _Ca, L_ was much slower in the presence of NaHS. The effect of NaHS induced a shift in the kinetics of recovery of I _Ca, L_ from inactivation; and the I/I _max_ values of the NaHS perfused group significantly decreased in comparison with that of the control, as the interval of pulses increased stepwise from 20 to 200 ms in 20 ms increments.

### Effects of sulfhydryl-modifying reagents (DM and DTT) on cardiomyocyte L-type Ca^2+^ channels

Fig. S1A shows the electrophysiological effects of 100 µmol/L DM on I_Ca, L_ in a control cardiomyocyte group (curve 1) compared with the 100 µmol/L DM-treated group (curve 2). The peak I _Ca, L_ elicited by test pulses from −40 to 0 mV was plotted over a recording time course of a total of 14 min. In the DM-treated (8 min) group, the peak I _Ca, L_ markedly decreased by 48.67±5.05% (n = 6, *P*<0.05) compared with the control group. A rapid depression took place at the beginning of the 5 min of extracellular application of 100 µmol/L DM, while the steady inhibitory effect of DM on I _Ca, L_ developed from 7 min after the drug perfusion.

Pooled data of the DTT-treated group and the controls are shown in Fig. S1B. It was found that either 1 mmol/L or 5 mmol/L DTT elicited almost no significant decrease in peak I _Ca, L_. However, application of either 1 mmol/L or 5 mmol/L DTT had a very slow and slightly decreasing effect on I _Ca, L_ in a time-dependent manner when the perfusion time was longer than 6 min.

Although DTT had no direct effect on L type calcium channels, the inhibition of DM on peak I _Ca, L_ could be abolished completely by bath application of DTT. As shown in Fig. S1C, after application of DM for 8 min, the peak Ca^2+^ current decreased to the lowest value; however, when 5 mmol/L DTT was applied, the peak Ca^2+^ current gradually increased. The mean peak amplitude of calcium current obtained from perfusion with 5 mmol/L DTT for 5 min increased from 67.12±4.86% to 83.91±4.92% of baseline (n = 6, *P*<0.01). Thus, it seems that the DTT has a dissociating effect on the decrease in the L-type calcium currents induced by DM.

### Sulfhydryl modifiers impact NaHS-induced inhibition of L-type calcium currents in cardiomyocytes

**Figure 3:**
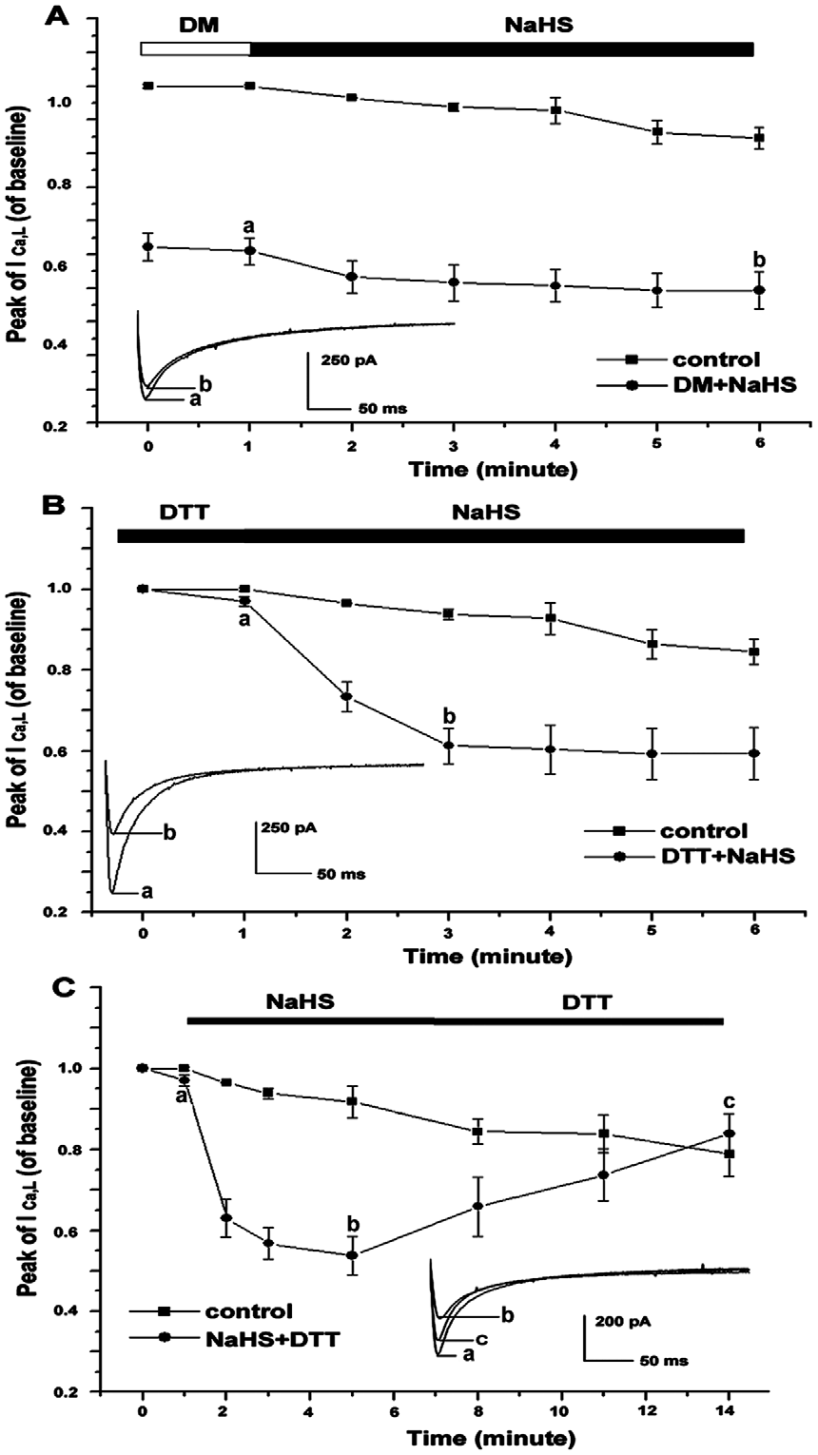
Effects of H_2_S donor on I _L, Ca_ modified by DM and DTT. A: DM significantly reduced the peak amplitude of I _Ca, L_ in cardiomyocytes, and the decrease by pre-treated with DM was basically constant and time-independent from the beginning through the final time point of 1 mmol/L NaHS perfusion period. B: DTT did not change the peak I _Ca L_, while removal of DTT by washing out with a 1 mmol/L NaHS-containing solution could decrease the peak I _Ca, L_ significantly. C: NaHS induced a decrease in the peak I _Ca, L_, and this decrease promptly reversed by DTT.

To examine if the NaHS-induced inhibitory effect on cardiac function in isolated perfused rat hearts depends on protein sulfhydryl groups, we used DM, an oxidizing sulfhydryl modifying substance, and DTT, a reducing sulfhydryl modifying regent, in this part of the experiment. Fig. 3A and Fig. 3B show the effect of NaHS on the peak I _Ca, L_ of L-type calcium channels of cardiomyocytes pre-treated with DM and DTT, respectively. We found that a significant decrease in peak amplitude of I _Ca, L_ could be reduced by pre-incubation with 100 µmol/L DM for 10 min, and the decrease in peak amplitude of I _Ca, L_ in cardiomyocytes pre-treated by DM was basically constant and time-independent from the beginning through the final time point of 1 mmol/L NaHS perfusion period (beginning time point: 45.38±4.01%, end time point: 45.43±5.04%, n = 6, *P*>0.05), respectively, compared with controls. The above data suggested that the state favoring formation of protein disulfide bonds of cysteines blocked DM- or H_2_S donor (NaHS)-induced inhibition of L-type calcium currents.

Furthermore, we found that the reduction of sulfhydryl with DTT did not change the peak I _Ca L_, since the peak I _Ca, L_ of cardiomyocytes pre-treated with 1 mmol/L DTT for 10 min was 97±1.24% of the controls (*P*>0.05). Removal of DTT by washing with a 1 mmol/L NaHS-containing solution resulted to a significant decrease in peak I _Ca, L_ up to 65.3±6.06% of the control values (n = 6, *P*<0.05).

Fig. 3C showed that the NaHS induced a decrease in the peak I _Ca, L_, and this decrease may be promptly reversed by DTT. The peak of I _Ca, L_ was 97±1.44%, 58.58±4.86% and 106.44±4.92% of the control, respectively, from the beginning until the end time points of perfusion with 1 mmol/L NaHS, as well as during the period of washing with 5 mmol/L DTT. Thus, the decrease in peak I _Ca, L_ induced by NaHS depended on the state of the free sulfhydryl group. That is, NaHS affected L- type calcium channels with the free sulfhydryl group but not with the disulfide bonded cysteines on the L-type calcium channels.

### Effects of NaHS on the free sulfhydryl groups of L-type calcium channel in H9C2 cells

**Figure 4:**
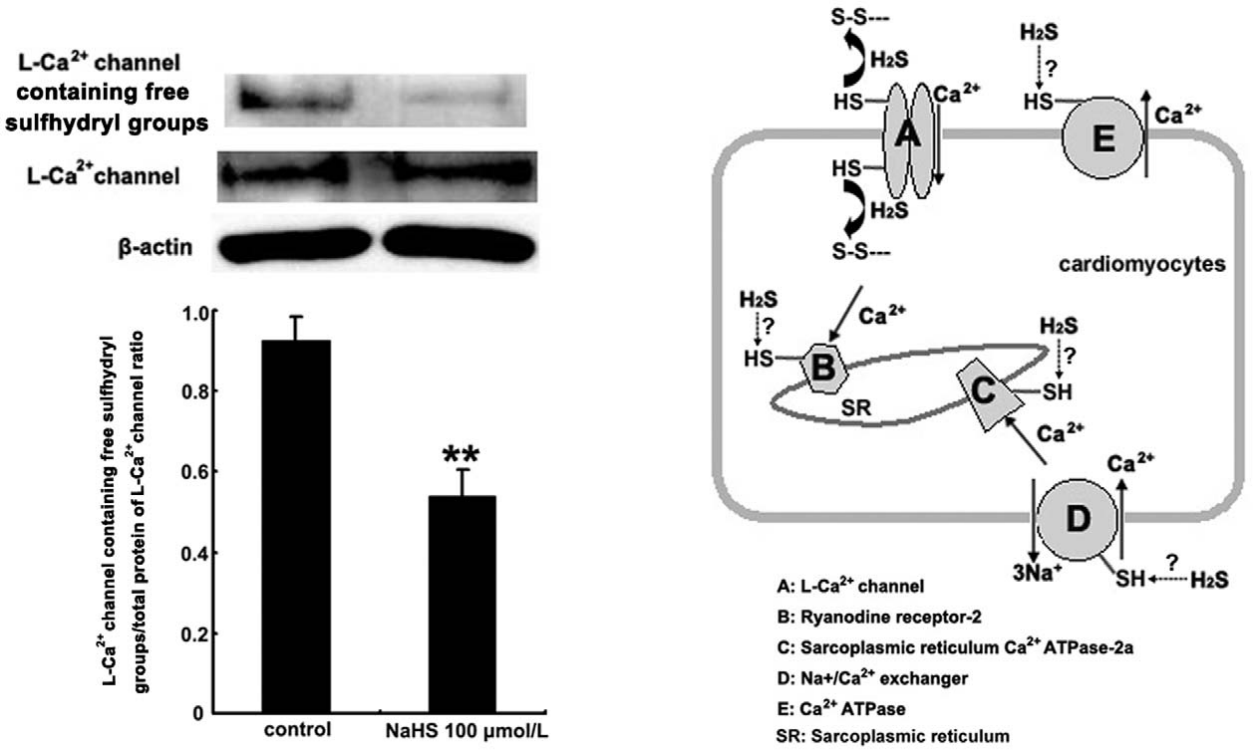
Effects of NaHS on the free sulfhydryl groups of L-type calcium channel in H9C2 cells, and a schematic picture showing L-type calcium channel and the other protein molecules involved in myocardial contraction that might react with H_2_S with their sulfhydryl groups. In the NaHS group, the L-type calcium channel containing free sulfhydryl groups/total protein of L-type calcium channel ratio in H9C2 cells decreased obviously, compared with that of the control group. ^**^ *P*<0.01 vs control group.

**Figure 5:**
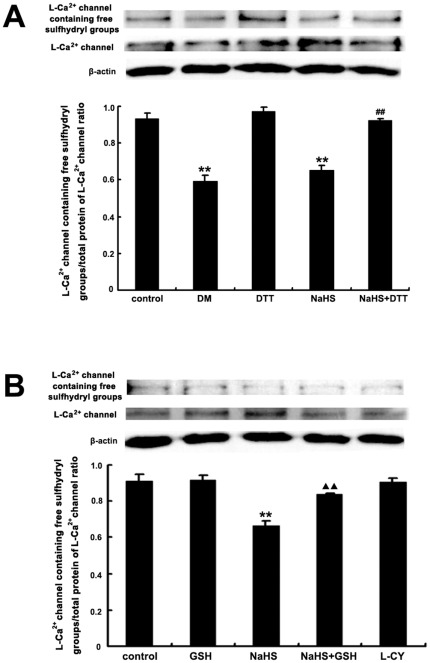
Effects of NaHS and sulfhydryl modifiers on the free sulfhydryl groups of L-type calcium channel in H9C2 cells. A: In NaHS and DM group, the L-type calcium channel containing free sulfhydryl groups/total protein of L-type calcium channel ratio in H9C2 cells reduced obviously, compared with that of the control group. ^**^ *P*<0.01 vs control group. In the NaHS+DTT group, the L-type calcium channel containing free sulfhydryl groups/total protein of L-type calcium channel ratio in H9C2 cells was reversed significantly, compared with that of the NaHS group. ^##^ *P*<0.01 vs NaHS group. B: In NaHS group, the L-type calcium channel containing free sulfhydryl groups/total protein of L-type calcium channel ratio in H9C2 cells reduced obviously, compared with that of the control group. ^**^ *P*<0.01 vs control group. Compared with that of NaHS group, the L-type calcium channel containing free sulfhydryl groups/total protein of L-type calcium channel ratio in NaHS+GSH group was reversed significantly. ^▴▴^ *P*<0.01 vs NaHS group.

To demonstrate if H_2_S targeted sulfhydryl groups in the L-type calcium channels in rat cardiomyocytes, we detected the ratio of L-type calcium channel containing free sulfhydryl groups to total protien of L-type calcium channel in H9C2 cells incubated with 100 µmol/L NaHS by using Western blot. In the NaHS-treated group and the DM-treated group, the ratio of L-type calcium channel containing free sulfhydryl groups to total protein L-type calcium channel in H9C2 cells decreased obviously, compared with that of the control group (*P*<0.01, Fig. 4 and 5). In the NaHS+DTT treated group, however, the decreased ratio of L-type calcium channel containing free sulfhydryl groups to total L-type calcium channel protein in H9C2 cells was significantly reversed, compared with that of the NaHS group (*P*<0.01, Fig. 4 and 5A). Additionally, compared with that of NaHS group, the decraesed ratio of L-type calcium channel containing free sulfhydryl groups to total L-type calcium channel protein in H9C2 cells was also significantly reversed in GSH+NaHS group (*P*<0.01, Figure 5B).

## Discussion

The results showed that the H_2_S donor inhibited the I _Ca, L_ in cardiomyocytes, which is accordant to the previous results [6]. It was reported that H_2_S might directly inhibit voltage-gated Ca^2+^ channels in vascular smooth muscle by Zhao et al. in 2002 [14], and it was also demonstrated that H_2_S was a novel inhibitor of L-type calcium channels in cardiomyocytes through electrophysiological measurements by Sun, et al. in 2009 [6]. Then, in 2011 Xu et al. found that the L-type Ca^2+^ channel agonist Bay K8644 could prevent from the electrophysiological effects of H_2_S by using a standard intracellular microelectrode technique [15]. The abovementioned results suggested that H_2_S could serve as an inhibitor of L-type calcium channels and the reduction in calcium influx might contribute to the functional effects of H_2_S [15]. DTT, a reductant which transforms disulfide bridges into sulfhydryl groups in cysteine-containing proteins, could markedly reverse the H_2_S donor-induced inhibition of I _Ca, L_ in cardiomyocytes. However, in the presence of DM, an oxidant which transforms sulfhydryl groups into disulfide bridges, NaHS could not alter cardiac function and L-type calcium currents. Furthermore, we found that after we treated the isolated rat heart or the cardiomycytes with DTT, NaHS could markedly alter cardiac function in isolated perfused heart and L-type calcium currents in the cardiomyocytes. Thus, the results suggest that the decrease in peak I _Ca, L_ induced by NaHS depend on the state of free sulfhydryl group. NaHS can affect L-type calcium channels with the sulfhydryl group, but it cannot affect those with the disulfide bonded cysteine groups.

H_2_S is determined to be a gasotransmitter alongside with NO and CO since it is a colorless, water-soluble and lipid-soluble gas of small size and can be endogenously generated and regulated by specific enzymes. It has broad physiological effects, but its relaxing effect on the cardiovascular system is unique [16]. Our *in vitro* study demonstrated that H_2_S can generate negative inotropic effects on the isolated rat heart. For example, NaHS (10^−6^–10^−3^ mol/L) could inhibit the ventricular contractile function in a concentration-dependent manner, and NaHS of 10^−3^ mol/L inhibited the coronary perfusive flow (CPF) and altered the left ventricular pressure. Administration of NaHS to the rat heart induced a transient negative cardiac inotropic effect and a decrease in central venous pressure [17]. Consistent with the results mentioned above, the present study confirmed that perfusion of NaHS at a 100 µmol/L concentration significantly decreased LV ±dp/dt_max_ and ΔLVP without changing heart rate and CPF.

In accordance with the inhibition of ventricular contractile function by the administration of NaHS, NaHS also inhibited I _Ca, L_ in rat cardiomyocytes in a concentration-dependent manner, but without changing the channel dynamic characteristics (i.e., shift in I–V relationship, activation and inactivation curves). The dynamic characteristics of resting, activation and inactivation states of L-type calcium channels could not be changed by H_2_S while the recovery curve was inhibited, suggesting that H_2_S could quickly occupy but then slowly dissociate from the L-type calcium channels. The entry of Ca^2+^ via the L-type calcium channels would trigger the opening of the calcium-releasing channels located in the calcium stores of the SR, and the increase in intracellular Ca^2+^ concentration would induce the contraction of cardiomyocytes. It has been reported [6] that H_2_S does not inhibit the caffeine-induced increase in intracellular Ca^2+^ concentration ([Ca^2+^]i). We considered that H_2_S induced a local decrease in [Ca^2+^]i by blocking the L-type calcium channels but not by the calcium-releasing channels of SR, and the decrease in [Ca^2+^]_i_ would lead to the attenuated contraction of cardiomyocytes. Our *in vivo* experiment gave the evidence that nifedipine pre-perfusion could inhibit the negative cardiac inotropic effect exerted by H_2_S. This result confirmed that the inhibition of ventricular contractile function by H_2_S was probably mediated by blocking the L-type calcium channels.

The substituted-cysteine accessibility method (SCAM) was widely used to elucidate the functions of ion channels [18]. The oxidation states of the sulfhydryl groups of the cysteine-containing peptides and proteins are critical to stabilization of its structure and function, and a special sulfhydryl modifier can localize functional regions of the molecule. Sulfhydryl reagents are crucial in SCAM. DTT is an effective sulfhydryl reductant that can reduce disulfide bonds regardless of inter-chain or intra-chain linkages [19]. DM is a commonly used sulfhydryl oxidizer since it can promote formation of a disulfide bridge between two cysteine residues when they are adjacent in the three-dimensional structure of a protein [20]. In the present study we found that the L-type Ca currents were decreased by 1 mmol/L DM, and the decrease could be reversed by 5 mmol/L DTT, while either 1 mmol/L or 5 mmol/L DTT had no direct effect on I _Ca, L_. These results suggest that the sulfhydryl groups on the L-type Ca^2+^ channels are important gate sites that can be directly modified by sulfhydryl reagents. L-type calcium channel on myocardiocytic membrane consists of a pore-forming α1c subunit and regulatory α2, δ and β subunits [21]. The α1c subunit which determines the basic electrophysiological properties and effect as a voltage sensor and receptor for antagonists/agonists has free sulfhydryl groups [22], while disulfide bonds are present between the α_2_ and δ subunits [23]. DM provides an oxidative environment which can form a disulfide bridge to stabilize the three-dimensional structure of the protein. Therefore, it can be proposed that the formation of disulfide bonds in the α_1_ subunit is the site affected by DTT. Studies on N-ethylmaleimide (NEM), chloramine-T (CL-T), 2, 2¢-dithiodipyridine (DTDP) and 2, 2¢-dithio-bis-5-nitropyridine (DTBNP) also showed a diminished effect on I _Ca, L_. Other results also indicated sulfhydryl reagents could directly act on the ion channel, since the effect was not due to either cAMP production or G-protein-coupled regulation of L-type Ca^2+^ channels [12].

The present results confirmed that I _Ca, L_ in the rat heart was inhibited by H_2_S, and the thiol oxidant DM was observed to cause a decrease in I _Ca, L_; and with pre-exposure to DM followed by perfusion with H_2_S, the Ca^2+^ current did not change compared with the control value. DTT had no direct effect on I _Ca, L_, although it could reverse the inhibition of I _Ca, L_ by NaHS. Since free sulfhydryl groups on the L-type Ca^2+^ channels are the gate sites, and they could be directly modified by hydrosulfuryl reagents, H_2_S would have no targeting site since DM would have already changed the oxidation state of the sulfhydryl groups of the L-type Ca^2+^ channels and formed a disulfide bridge between adjacent cysteine residues in the three-dimensional structure. If H_2_S targets on the crucial free-sulfhydryl groups on the Ca^2+^ channel and inhibits the L-type calcium current, the inter-chain disulfide bond linkages would be rapidly reduced by DTT, and therefore the inhibition would be reversed. Thus, H_2_S appears to function by activating a thiol oxidation mechanism that inhibits L-type Ca^2+^ channels.

To further demonstrate if H_2_S targeted the sulfhydryl groups in the L-type calcium channels in rat cardiomyocytes, we measured the ratio of L-type calcium channel containing free sulfhydryl groups to total L-type calcium channel protein in H9C2 cells incubated with H_2_S donor by Western blot. After treatment with H_2_S donor, the ratio of L-type calcium channel containing free sulfhydryl groups to total L-type calcium channel protein in H9C2 cells decreased obviously. However, the decreased ratio of L-type calcium channel containing free sulfhydryl groups to total L-type calcium channel protein in H9C2 cells was significantly reversed by a thiol reductant DTT. Additionally, it was also reversed by another thiol reductant GSH, suggesting that H_2_S could target the sulfhydryl group, decreasing the reduced thiol of L-Ca^2+^ channel in H9C2 cells, which could be reversed by thiol reductants.

We believe that the sulfhydryl groups on the cysteine-containing proteins may play an important mechanistic role in the biological effects of H_2_S on the cardiovascular system. Like L-type calcium channels, the sulfhydryl groups of ATP-sensitive potassium channels (K_ATP_ channels) also are the channel gate sites, and the vasodilating effect ascribed to H_2_S to open K_ATP_ channels has been elucidated. Endogenous H_2_S has been reported as a novel inhibitor to suppress the proliferation of vascular smooth muscle cells (VSMCs) through the mitogen-activated protein kinase (MAPK) pathway [24]. Previous research found that the MAPK/extracellular-signal-regulated kinase kinase 1, an upstream activator of the stress-activated protein kinase/c-Jun N-terminal kinase pathway, is directly inhibited by cysteine modification. Further studies are needed to reveal details of the substantial role for thiol modification of specific protein targets involved in the H_2_S-mediated biological effects.

## Supporting Information

Figure S1 **L-type Ca^2+^ current was affected by extracellularly applied sulfhydryl modifying reagents.** A: In the DM-treated group. The peak I _Ca, L_ markedly decreased, compared with the control group. A rapid depression took place at the beginning of the 5 min of extracellular application of 100 µmol/L DM, while the steady inhibitory effect of DM on I _Ca, L_ developed from 7 min after the drug perfusion. B: DTT elicited almost no significant decrease in peak I _Ca, L_. However, application of DTT had a very slow and slightly decreasing effect on I _Ca, L_ in a time-dependent manner when the perfusion time was longer than 6 min. C: DTT almost completely reversed the inhibition of DM on peak I _Ca, L_.
(TIF)

## References

[1] Tang C, Li X, Du J (2006). Hydrogen sulfide as a new endogenous gaseous transmitter in the cardiovascular system. Curr Vasc Pharmacol..

[2] Du JB, Zhang CY, Yan H (2006). A newly found gasotransmitter, hydrogen sulfide, in the pathogenesis of hypertension and other cardiovascular diseases. Curr Hypertens Rev..

[3] Ji Y, Pang QF, Xu G, Wang L, Wang JK (2008). Exogenous hydrogen sulfide postconditioning protects isolated rat hearts against ischemia-reperfusion injury. Eur J Pharmacol..

[4] Utpal S, Thomas PV, William MH, Munish K, Karni SM (2008). Cardioprotective role of sodium thiosulfate on chronic heart failure by modulating endogenous H_2_S generation. Pharmacology..

[5] Szentesi P, C Pignier C, M Egger M, Kranias EG (2004). Sarcoplasmic reticulum Ca^2+^ refilling controls recovery from Ca^2+^-induced Ca^2+^ release refractoriness in heart muscle. Circ Rec..

[6] Sun YG, Cao YX, Wang WW, Ma SF, Yao T (2008). Hydrogen sulphide is an inhibitor of L-type calcium channels and mechanical contraction in rat cardiomyocytes. Cardiovasc Res..

[7] Thompson RW, Valentine HL, Valentine WM (2003). Cytotoxic mechanisms of hydrosulfide anion and cyanide anion in primary rat hepatocyte cultures. Toxicology..

[8] Eghbal MA, Pennefather PS, O'Brien PJ (2004). H_2_S cytotoxicity mechanism involves reactive oxygen species formation and mitochondrial depolarisation. Toxicology..

[9] Smith RP, Abbanat RA (1966). Protective effect of oxidized glutathione in acute sulfide poisoning. Toxicol Appl Pharamcol..

[10] Chiamvimonvat N, O'Rourke B, Kamp TJ, Kallen RG, Hofmann F (1995). Functional consequences of sulfhydryl modification in the pore-forming subunits of cardiovascular Ca^2+^ and Na^+^ channels. Circ Res..

[11] Tanaka Y, Sasaki N, Tsuboi M, Miake J, Kinugawa T (1998). Sulfhydryl oxidation activates the cardiac ATP sensitive K^+^channel (IK_ATP_) via forming a disulfide bridge among cysteine residues of the pore: novel mechanism on activation of IK_ATP_ independent of cytosolic ATP level. Circulation..

[12] Yamaoka K, Yakehiro M, Yuki T, Fujii H, Seyama I (2000). Effect of sulfhydryl reagents on the regulatory system of the L-type Ca channel in frog ventricular myocytes. Pflugers Arch..

[13] Zhang ZH, Boutjdir M, ElSherif N (1994). Ketanserin inhibits depolarizationactivated outward potassium current in rat ventricular myocytes. Circ Res..

[14] Zhao W, Wang R (2002). H(2)S-induced vasorelaxation and underlying cellular and molecular mechanisms. Am J Physiol Heart Circ Physiol..

[15] Xu M, Wu YM, Li Q, Liu S, Li Q (2011). Electrophysiological effects of hydrogen sulfide on human atrial fibers. Chin Med J (Engl)..

[16] Pearson RJ, Wilson T, Wang R (2006). Endogenous hydrogen sulfide and the cardiovascular system-what's the smell all about? Clin Invest Med..

[17] Geng B, Yang J, Qi Y, Zhao J, Pang Y (2004). H_2_S generated by heart in rat and its effects on cardiac function. Biochem Biophys Res Commun..

[18] Perret P, Laube B, Schemmol L R, Betz H, Goeldner M (2002). Affinity labeling of cysteine-mutants evidences contact residues in modeled receptor binding sites. J Recept Signal Transduct Res..

[19] Kobayashi O, Matsui K, Minamiura N, Yamamoto T (1985). Effect of dithiothreitol on activity and protein structure of human urine urokinase. J Biochem..

[20] Liang H, Li X, Li S, Zheng MQ, Rozanski GJ (2008). Oxidoreductase regulation of Kv currents in rat ventricle. J Mol Cell Cardiol..

[21] Hullin R, Asmus F, Ludwig A, Hersel J, Boekstegers P (1999). Subunit expression of the cardiac L-type calcium channel is differentially regulated in diastolic heart failure of the cardiac allograft. Circulation..

[22] Gao TY, Bünemann M, Gerhardstein BL, Ma H, Hosey MMOL L (2000). Role of the C terminus of the α1C(CaV1.2) subunit in membrane targeting of cardiac l-type calcium channels. J Biol Chem..

[23] Wiser O, Trus M, Tobi D, Halevi S, Giladi E (1996). The α2/δ subunit of voltage sensitive Ca2+ channels is a single transmembrane extracellular protein which is involved in regulated secretion. FEBS..

[24] Cross JV, Templeton DJ (2004). Oxidative stress inhibits MEKK1 by site-specific glutathionylation in the ATP-binding domain. Biochem J..

