# Supplementary figures and images for: Hydrogen Sulfide Inhibits L-Type Calcium Currents Depending upon the Protein Sulfhydryl State in Rat Cardiomyocytes
Source: PLoS One. 2012 May 10;7(5):e37073. doi: 10.1371/journal.pone.0037073 (PMC3349658; doi:10.1371/journal.pone.0037073)

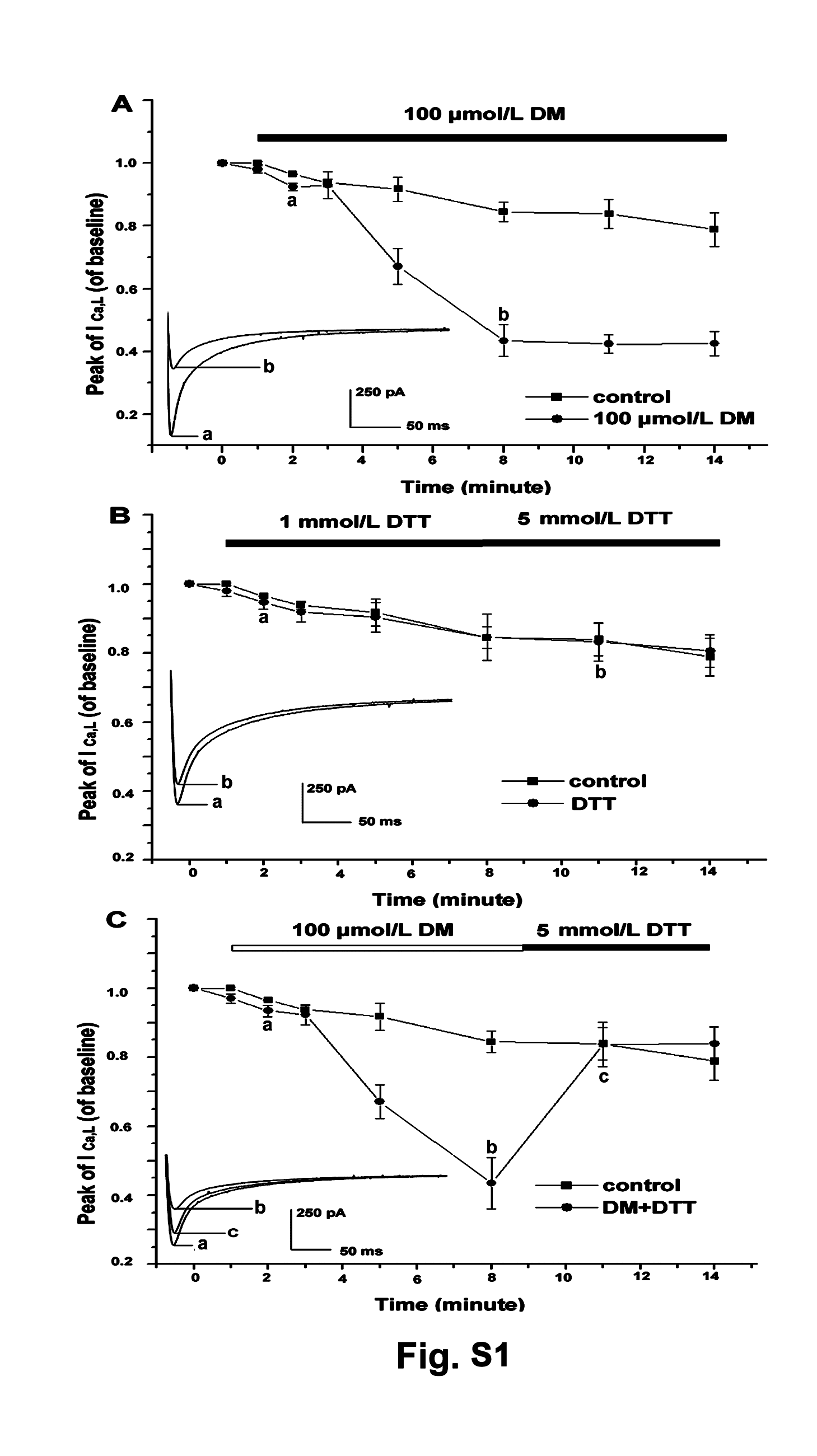

Supplement: Figure S1 — L-type Ca2+ current was affected by extracellularly applied sulfhydryl modifying reagents. A: In the DM-treated group. The peak I Ca, L markedly decreased, compared with the control group. A rapid depression took place at the beginning of the 5 min of extracellular application of 100 µmol/L DM, while the steady inhibitory effect of DM on I Ca, L developed from 7 min after the drug perfusion. B: DTT elicited almost no significant decrease in peak I Ca, L. However, application of DTT had a very slow and slightly decreasing effect on I Ca, L in a time-dependent manner when the perfusion time was longer than 6 min. C: DTT almost completely reversed the inhibition of DM on peak I Ca, L. (TIF) [file pone.0037073.s001.tif]
